# Supplementary material for: Light-driven activation of mitochondrial proton-motive force improves motor behaviors in a Drosophila model of Parkinson’s disease
Source: Commun Biol. 2019 Nov 22;2:424. doi: 10.1038/s42003-019-0674-1 (PMC6874642; doi:10.1038/s42003-019-0674-1)
Supplement: Supplementary file 1 — Supplemental Information [file 42003_2019_674_MOESM1_ESM.pdf]

Supplementary Figures

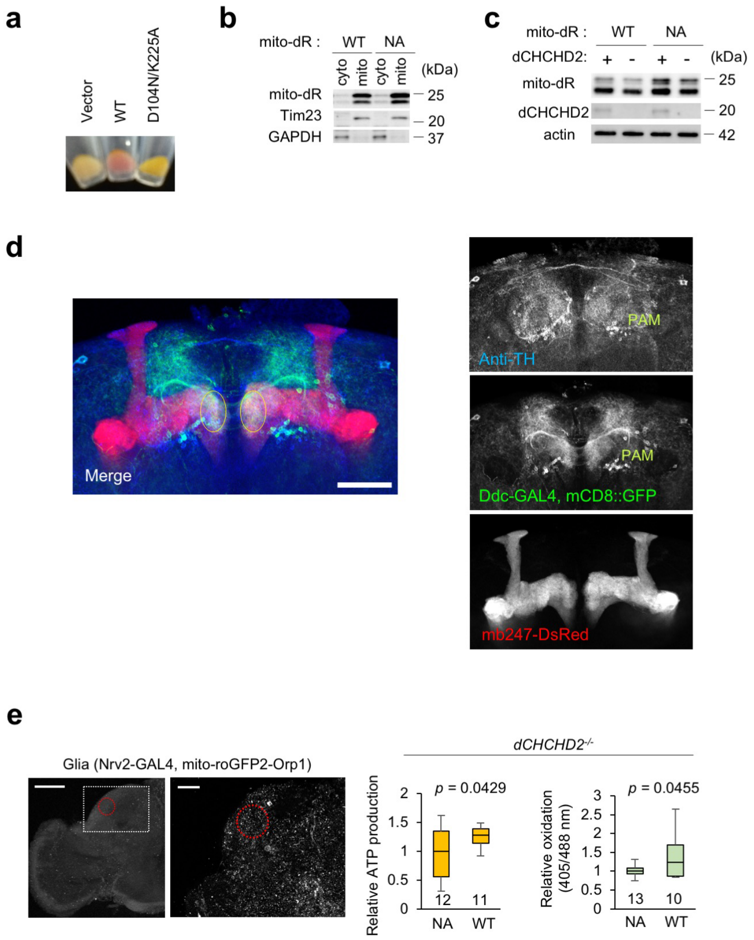

Supplementary Fig. 1. Effects of mito-dR on glial cells.
(**a**) Replacement of dR D104 and K225 with N and A results in loss of retinal-binding activity. dR WT, D104N/K225A (NA) mutant and an empty vector were expressed in *E. coli* in the presence of retinal, which resulted in purple pigmentation of dR due to retinal retention. The lack of retinal-binding ability in NA mutant was confirmed by the loss of pigmentation. (**b**) mito-dR is localized at mitochondria. $S2R^+$ cells were transfected with plasmids for WT dR or NA mutant harboring a mitochondrial targeting sequence (mito-dR). Cytosolic (cyto) and mitochondrial (mito) proteins were fractionated and subjected to western blotting. Tim23 and GAPDH are mitochondrial and cytosolic markers, respectively. (**c**) Generation of mito-dR transgenic flies. Expression of mito-dR on wild-type (w^{1118}) and *dCHCHD2*$^{-/-}$ backgrounds was confirmed using brain tissues. Actin served as a loading control. (**d**) DA neuron terminals project to the mushroom bodies. Anterior part of adult brain containing the mushroom bodies (red). The PAM neuron cell bodies and DA terminals stained with anti-TH (blue) were also visualized by mCD8::GFP (green). mCD8::GFP was driven by the *Ddc-GAL4* driver. The mushroom bodies were visualized by *mb247-DsRed*. Scale bar = 50 µm. (**e**) mito-dR WT or NA along with the ATP biosensor ATeam (left graph) or mitochondrial H_2O_2 biosensor mito-roGFP2-Orp1 (right graph) was expressed in the cortex glia, astrocyte-like glia and ensheathing glia of *dCHCHD2*$^{-/-}$ flies. The glial cells circled by dashed red lines (grayscale images, mito-roGFP2-Orp1 at 488 nm, scale bars = 100 [left] and 25 [right] µm) were analyzed. Graphs represent relative values normalized with NA, and statistical significance was assessed using two-tailed Student's *t*-test. The image on the right is a higher magnification of the area boxed in the left image.

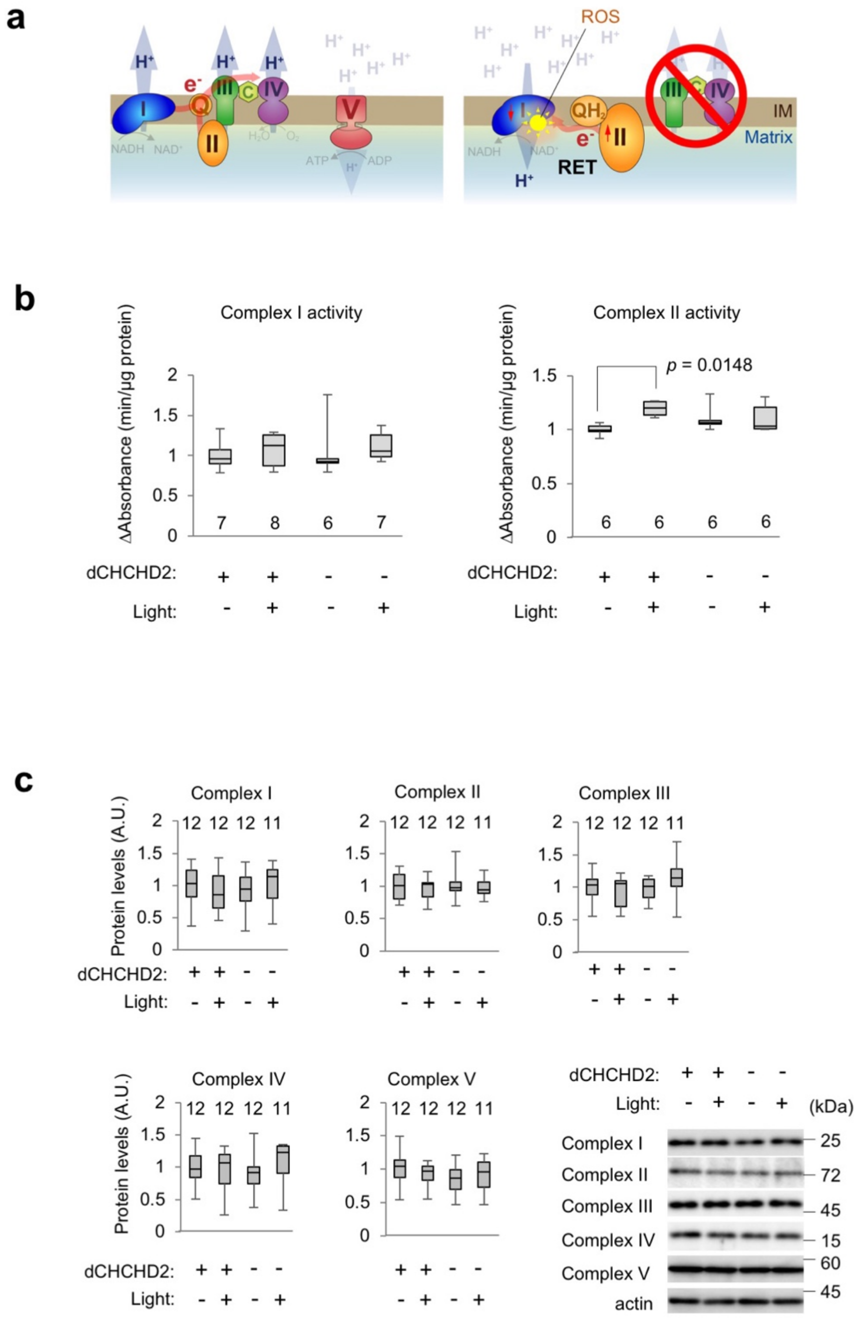

Supplementary Fig. 2. dR does not affect the expression of mitochondrial respiratory complexes. (**a**) During forward electron transfer, coenzyme Q (Q) receives electrons (e^-) from complexes I and II, which are further transferred to complexes III and IV (left). The conditions of high Δp and QH_2/Q ratio induce reverse electron transport (RET) from complex II to complex I. RET produces higher ROS in complex I, resulting in complex I degradation (right). (**b**) Respiratory complex I and II activity. Mitochondria isolated from 30-day-old male fly heads were subjected to spectrophotometric analysis of respiratory chain enzyme activities. n = 6-8 biological replicates, Tukey-Kramer test. (**c**) Boxplots represent quantification of OXPHOS protein levels by western blot analysis using anti-NDUFS3 (for Complex I), anti-SDHA (for Complex II), anti-UQCRC1 (for Complex III), anti-COX IV (for Complex IV) and anti-ATP5A (for Complex V). Brain tissues from 30-day-old male flies were analyzed. No differences were observed by Dunnett's test (n = 11-12 flies).
Transgenes were driven by *Da-GAL4*.

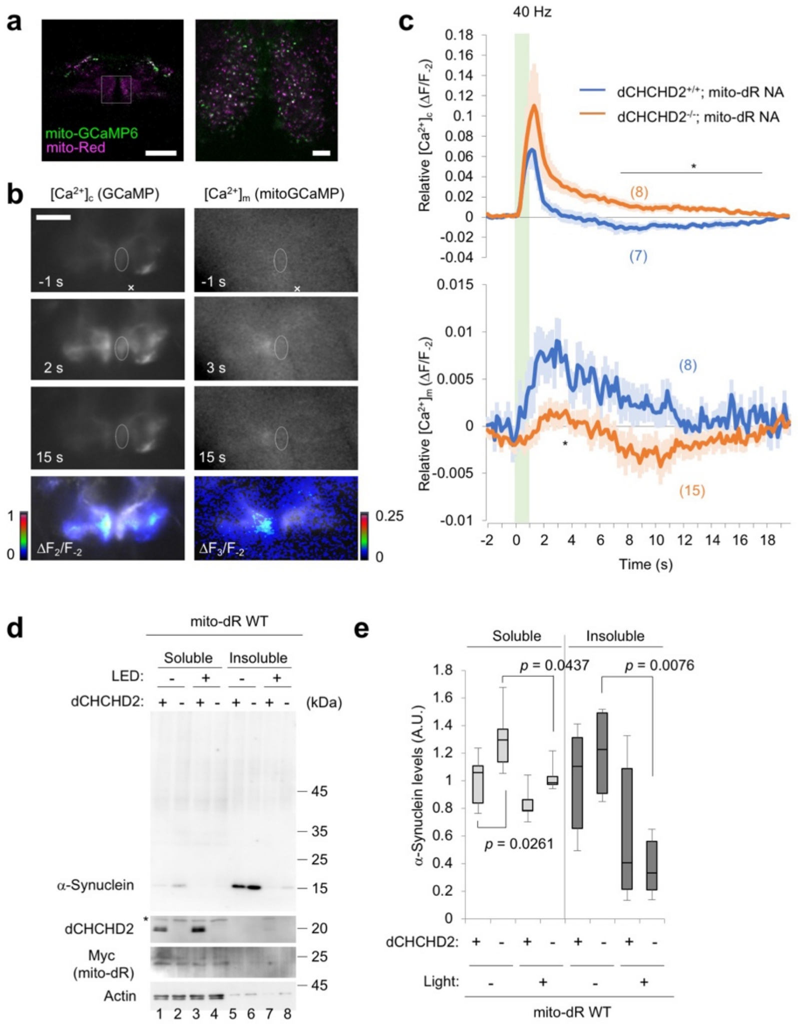

Supplementary Fig. 3. Ca^{2+}-buffering activity and α-synuclein turnover are impaired by dCHCHD2 loss.

(**a**) mito-GCaMP6 is localized in the mitochondria. mito-GCaMP6 and mito-DsRed were coexpressed in the PAM neurons. The region containing the nerve terminals of PAM neurons is surrounded by a dashed box in the left panel. A high magnification image of the boxed region is shown on the right. Scale bars = 50 μm (left), 5 μm (right). (**b**) Dynamics of $[Ca^{2+}]_c$ and $[Ca^{2+}]_m$ in the fly brain expressing *mb247-DsRed* were estimated by GCaMP6f and mito-GCaMP6, respectively. Dashed circles and crosses indicate the recorded regions and stimulated regions (upper side of antennal lobes), respectively. mb247-DsRed signals to visualize the mushroom bodies (grayscale) were overlaid with ΔF/F signals (rainbow scales, bottom). Scale bar = 50 μm. (**c**) Measurement of $[Ca^{2+}]_c$ and $[Ca^{2+}]_m$ in the nerve terminals of *dCHCHD2*$^{+/+}$ and *dCHCHD2*$^{-/-}$ PAM neurons. GCaMP6f or mito-GCaMP6 along with nonfunctional mito-dR NA was expressed in PAM neurons, and the $[Ca^{2+}]$ measurement was performed as in Fig. 3a. * $p < 0.05$, two-tailed Student's *t*-test in the integrated value of every s. The numbers of samples analyzed are described in parentheses of the graphs. (**d, e**) mito-dR activation by light irradiation suppresses α-synuclein accumulation by dCHCHD2 loss. (**d**) Sarkosyl-soluble and insoluble brain extracts from flies expressing mito-dR WT with or without light irradiation were analyzed by western blotting with the indicated antibodies. An asterisk indicates nonspecific bands. (**e**) Boxplots represent quantitative western blot analysis of α-synuclein. The band intensity of dCHCHD2$^{+/+}$, mito-dR WT without light in each fraction was set to 1. Brain tissues from 30-day-old male flies were analyzed. A.U., arbitrary units. n = 6 biological replicates, Tukey-Kramer test.
Transgenes were driven by *R58E02-GAL4* (**a-c**) or *Ddc-GAL4* (**d, e**).

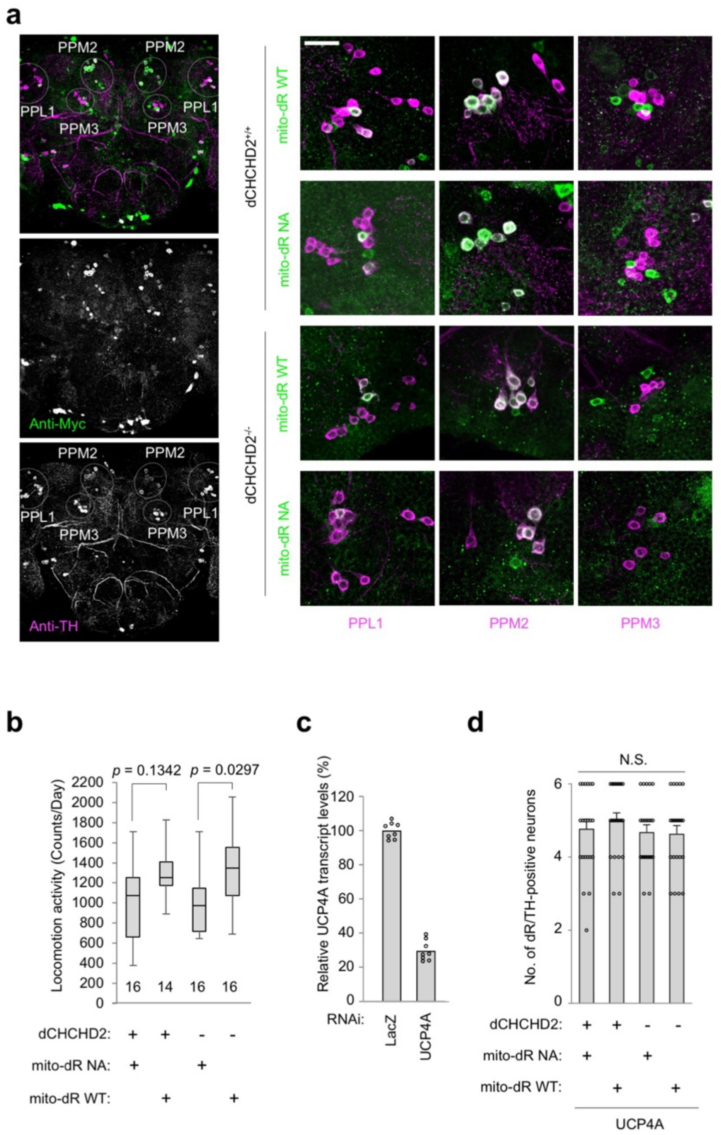

Supplementary Fig. 4. Spontaneous locomotor activity of *dCHCHD2$^{-/-}$* flies was activated by mito-dR.

(**a**) (Left) Whole brain images from the posterior side containing the PPL1, PPM2 and PPM3 DA neuron clusters of normal flies expressing Myc-tagged mito-dR WT. Scale bar = 100 µm. (Right) Representative images of DA neurons expressing mito-dR with the indicated genotypes, related to Fig. 4a. The brain tissues of 30-day-old male files were stained with anti-Myc (green) and anti-TH (red) antibodies. Scale bar = 20 µm. Note that mito-dR is expressed in only a few and rare TH-positive neurons in PPL1 and PPM3 clusters, respectively, because the *Ddc-GAL4* driver does not cover all TH-positive neurons. (**b**) The activity of individual flies in polycarbonate tubes was quantified with the *Drosophila* Activity Monitoring (DAM) system as passage by an infrared beam bisecting the tube. n = 14-16 flies per genotype for a day, Tukey-Kramer test. Transgenes were driven by *Ddc-GAL4*. (**c**) Knockdown efficiency of UCP4A RNAi. UCP4A RNAi (VDRC ID: 102571) and control LacZ RNAi were driven by *elav-GAL4*. RT-qPCR was performed using total RNA from the whole brain of flies raised at 28°C. *UCP4A* transcription levels normalized with housekeeping *rp49* were graphed (mean ± s.e.m.). Two independent samples from RNA extracts with 6 flies per genotype. (**d**) Dopaminergic expression of UCP4A partially rescued DA neuron loss in *dCHCHD2$^{-/-}$* flies. The numbers of dR-positive PPM2 cluster DA neurons in 30-day-old flies are graphed (mean ± s.e.m.). n = 22 flies per genotype. N.S., not significant by Tukey-Kramer test.

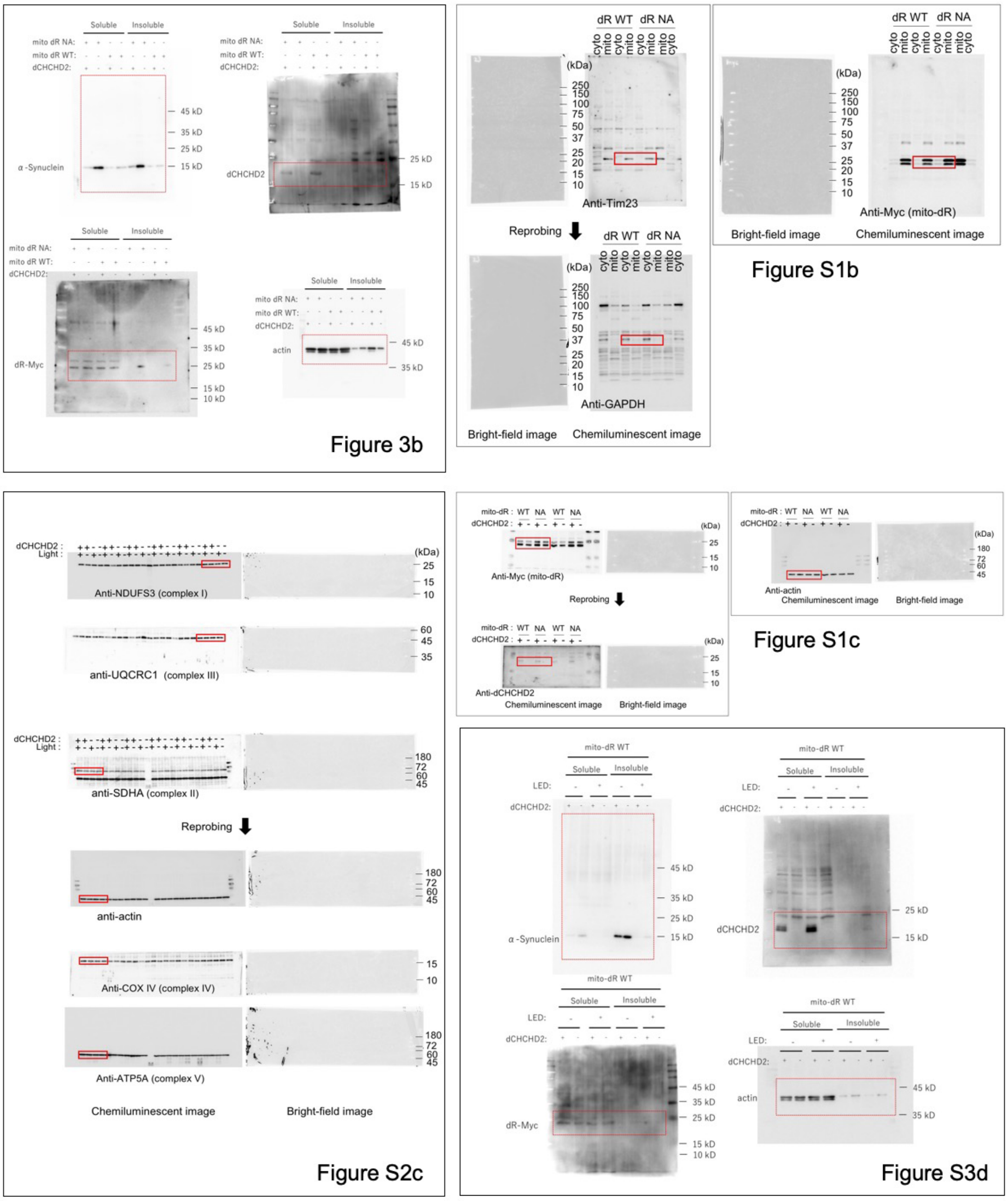

Supplementary Fig. 5. Uncropped images of western blots used in Figure 3b and Supplementary Figure 1 - 3.

Supplementary Notes

Fly genotypes used in this study

Fig.1c
+/Y; UAS-mitoGFP/+; D42-Gal4/UAS-mito-dR WT (mito-dR WT; dCHCHD2 +)
CG5010^{null}*/Y; UAS-mitoGFP/+; D42-Gal4/UAS-mito-dR WT* (mito-dR WT; dCHCHD2 -)
+/Y; UAS-mitoGFP/+; D42-Gal4/UAS-mito-dR NA (mito-dR NA; dCHCHD2 +)
CG5010^{null}*/Y; UAS-mitoGFP/+; D42-Gal4/UAS-mito-dR NA* (mito-dR NA; dCHCHD2 -)

Fig. 1d
+/Y; +/+; Da-Gal4/UAS-mito-dR WT (dCHCHD2 +)
CG5010^{null}*/Y; +/+; Da-Gal4/UAS-mito-dR WT* (dCHCHD2 -)

Fig. 1e, f
+/Y; UAS-AT1.03NL/+; Ddc-Gal4/UAS-mito-dR NA (mito-dR NA; dCHCHD2 +)
+/Y; UAS-AT1.03NL/+; Ddc-Gal4/UAS-mito-dR WT (mito-dR WT; dCHCHD2 +)
CG5010^{null}*/Y; UAS-AT1.03NL/+; Ddc-Gal4/UAS-mito-dR NA* (mito-dR NA; dCHCHD2 -)
CG5010^{null}*/Y; UAS-AT1.03NL/+; Ddc-Gal4/UAS-mito-dR WT* (mito-dR WT; dCHCHD2 -)

Fig. 2a-d, g, h
+/Y; +/+; Da-Gal4/UAS-mito-dR WT (mito-dR WT; dCHCHD2 +)
CG5010^{null}*/Y; +/+; Da-Gal4/UAS-mito-dR WT* (mito-dR WT; dCHCHD2 -)
+/Y; +/+; Da-Gal4/UAS-mito-dR NA (mito-dR NA; dCHCHD2 +)
CG5010^{null}*/Y; +/+; Da-Gal4/UAS-mito-dR NA* (mito-dR NA; dCHCHD2 -)

Fig. 2e, f
+/Y; UAS-mito-roGFP2-Orp1/+; Ddc-Gal4/UAS-mito-dR WT (dCHCHD2 +)
CG5010^{null}*/Y; UAS-mito-roGFP2-Orp1/+; Ddc-Gal4/UAS-mito-dR WT* (dCHCHD2 -)

Fig. 3a
(Upper graph)
+/Y; UAS-GCaMP6f/+; R58E02-Gal4/UAS-mito-dR WT (dCHCHD2$^{+/+}$; mito-dR WT)
CG5010^{null}*/Y; UAS-GCaMP6f/+; R58E02-Gal4/ UAS-mito-dR WT* (dCHCHD2$^{-/-}$; mito-dR WT)
CG5010^{null}*/Y; UAS-GCaMP6f/+; R58E02-Gal4/ UAS-mito-dR NA* (dCHCHD2$^{-/-}$; mito-dR NA)

(Lower graph)
+/Y; +/+; R58E02-Gal4, UAS-mito-GCaMP6/UAS-mito-dR WT (dCHCHD2$^{+/+}$; mito-dR WT)
CG5010^{null}*/Y; +/+; R58E02-Gal4, UAS-mito-GCaMP6/UAS-mito-dR WT* (dCHCHD2$^{-/-}$; mito-dR WT)
CG5010^{null}*/Y; +/+; R58E02-Gal4, UAS-mito-GCaMP6/UAS-mito-dR NA* (dCHCHD2$^{-/-}$; mito-dR NA)

Fig. 3b, c
+/Y; UAS-α-Synuclein LP2/+; Ddc-Gal4/UAS-mito-dR NA (mito-dR NA; dCHCHD2 +)
+/Y; UAS-α-Synuclein LP2/+; Ddc-Gal4/UAS-mito-dR WT (mito-dR WT; dCHCHD2 +)
CG5010^{null}*/Y; UAS-α-Synuclein LP2/+; Ddc-Gal4/UAS-mito-dR NA* (mito-dR NA; dCHCHD2 -)
CG5010^{null}*/Y; UAS-α-Synuclein LP2/+; Ddc-Gal4/UAS-mito-dR WT* (mito-dR WT; dCHCHD2 -)

Fig. 4a
+/Y; +/+; Ddc-Gal4/UAS-mito dR WT (mito-dR WT; dCHCHD2 +)
+/Y; +/+; Ddc-Gal4/UAS-mito-dR NA (mito-dR NA; dCHCHD2 +)
CG5010^{null}*/Y; +/+; Ddc-Gal4/UAS-mito-dR WT* (mito-dR WT; dCHCHD2 -)
CG5010^{null}*/Y; +/+; Ddc-Gal4/UAS-mito-dR NA* (mito-dR NA; dCHCHD2 -)

Fig. 4b
+/Y; +/+; Da-Gal4/UAS-mito-dR WT (dCHCHD2 +)
CG5010^{null}*/Y; +/+; Da-Gal4/UAS-mito dR WT* (dCHCHD2 -)

Fig. 4c
+/Y; +/+; Ddc-Gal4/UAS-mito-dR WT (WT; dCHCHD2 +)
CG5010^{null}*/Y; +/+; Ddc-Gal4/UAS-mito-dR WT* (WT; dCHCHD2 -)
+/Y; +/+; Ddc-Gal4/UAS-mito-dR NA (NA; dCHCHD2 +)
CG5010^{null}*/Y; +/+; Ddc-Gal4/UAS-mito-dR NA* (NA; dCHCHD2 -)

Fig. 4d, e
CG5010^{null}*/Y; UAS-LacZ RNAi/+; Ddc-Gal4/UAS-mito-dR WT* (LacZ RNAi; WT)
CG5010^{null}*/Y; UAS-LacZ RNAi/+; Ddc-Gal4/UAS-mito-dR NA* (LacZ RNAi; NA)
CG5010^{null}*/Y; UAS-UCP4A RNAi/+; Ddc-Gal4/UAS-mito-dR WT* (UCP4A RNAi; WT)
CG5010^{null}*/Y; UAS-UCP4A RNAi/+; Ddc-Gal4/UAS-mito-dR NA* (UCP4A RNAi; NA)

Supplementary Fig. 1c
+/Y; +/+; Da-Gal4/UAS-mito-dR WT (mito-dR WT; dCHCHD2 +)
CG5010^{null}*/Y; +/+; Da-Gal4/UAS-mito-dR WT* (mito-dR WT; dCHCHD2 -)
+/Y; +/+; Da-Gal4/UAS-mito-dR NA (mito-dR NA; dCHCHD2 +)
CG5010^{null}*/Y; +/+; Da-Gal4/UAS-mito-dR NA* (mito-dR NA; dCHCHD2 -)

Supplementary Fig. 1d
mb247-DsRed/UAS-mCD8::GFP; Ddc-Gal4/+

Supplementary Fig. 1e
CG5010^{null}*/Y; nrv2-Gal4/UAS-AT1.03NL; UAS-mito-dR NA/+* (ATP: NA)
CG5010^{null}*/Y; nrv2-Gal4/UAS-AT1.03NL; UAS-mito-dR WT/+* (ATP: WT)
CG5010^{null}*/Y; nrv2-Gal4/UAS-mito-roGFP2-Orp1; UAS-mito-dR NA/+* (Oxidation: NA)
CG5010^{null}*/Y; nrv2-Gal4/UAS-mito-roGFP2-Orp1; UAS-mito-dR WT/+* (Oxidation: WT)

Supplementary Fig. 2b, c
+/Y; +/+; Da-Gal4/UAS-mito-dR WT (dCHCHD2 +)
CG5010^{null}*/Y; +/+; Da-Gal4/UAS-mito-dR WT* (dCHCHD2 -)

Supplementary Fig. 3a
+/Y; UAS-mitoGCaMP6/+; R58E02-Gal4/UAS-mitoDsRed

Supplementary Fig. 3b
+/Y; UAS-GCaMP6f/+; R58E02-Gal4/UAS-mitoDsRed (GCaMP)
+/Y; +/+; R58E02-Gal4, UAS-mito-GCaMP6/UAS-mitoDsRed (mito-GCaMP)

Supplementary Fig. 3c
(Upper graph)
+/Y; UAS-GCaMP6f/+; R58E02-Gal4/UAS-mito-dR NA (dCHCHD2$^{+/+}$; mito-dR NA)
CG5010^{null}*/Y; UAS-GCaMP6f/+; R58E02-Gal4/UAS-mito-dR NA* (dCHCHD2$^{-/-}$; mito-dR NA)

(Lower graph)
+/Y; +/+; R58E02-Gal4, UAS-mito-GCaMP6/UAS-mito-dR NA (dCHCHD2$^{+/+}$; mito-dR NA)
dCHCHD2^{null}*/Y; +/+; R58E02-Gal4, UAS-mito-GCaMP6/UAS-mito-dR NA* (dCHCHD2$^{-/-}$; mito-dR NA)

Supplementary Fig. 3d, e

+/Y; UAS-α-Synuclein LP2/+; Ddc-Gal4/UAS-mito-dR WT (mito-dR WT; dCHCHD2 +)
CG5010^{null}/Y; UAS-α-Synuclein LP2/+; Ddc-Gal4/UAS-mito-dR WT (mito-dR WT; dCHCHD2 -)

Supplementary Fig. 4a
+/Y; +/+; Ddc-Gal4/UAS-mito-dR WT (dCHCHD2$^{+/+}$; mito-dR WT)
+/Y; +/+; Ddc-Gal4/UAS-mito-dR NA (dCHCHD2$^{+/+}$; mito-dR NA)
CG5010^{null}/Y; +/+; Ddc-Gal4/UAS-mito-dR WT (dCHCHD2$^{-/-}$; mito-dR WT)
CG5010^{null}/Y; +/+; Ddc-Gal4/UAS-mito-dR NA (dCHCHD2$^{-/-}$; mito-dR NA)

Supplementary Fig. 4b
+/Y; +/+; Ddc-Gal4/UAS-mito-dR NA (mito-dR NA; dCHCHD2 +)
+/Y; +/+; Ddc-Gal4/UAS-mito-dR WT (mito-dR WT; dCHCHD2 +)
CG5010^{null}/Y; +/+; Ddc-Gal4/UAS-mito-dR NA (mito-dR NA; dCHCHD2 -)
CG5010^{null}/Y; +/+; Ddc-Gal4/UAS-mito-dR WT (mito-dR WT; dCHCHD2 -)

Supplementary Fig. 4c
elav-Gal4/Y; UAS-LacZ RNAi/+; +/+ (LacZ RNAi)
elav-Gal4/Y; UAS-UCP4A RNAi/+; +/+ (UCP4A RNAi)

Supplementary Fig. 4d
+/Y; +/+; Ddc-Gal4/UAS-mito-dR NA, UAS- UCP4A-3×HA (mito-dR NA; dCHCHD2 +)
+/Y; +/+; Ddc-Gal4/UAS-mito-dR WT, UAS-UCP4A-3×HA (mito-dR WT; dCHCHD2 +)
CG5010^{null}/Y; +/+; Ddc-Gal4/UAS-mito-dR NA, UCP4A-3×HA (mito-dR NA; dCHCHD2 -)
CG5010^{null}/Y; +/+; Ddc-Gal4/UAS-mito-dR WT, UCP4A-3×HA (mito-dR WT; dCHCHD2 -)
