# Supplementary material for: Light-driven activation of mitochondrial proton-motive force improves motor behaviors in a Drosophila model of Parkinson’s disease
Source: Commun Biol. 2019 Nov 22;2:424. doi: 10.1038/s42003-019-0674-1 (PMC6874642; doi:10.1038/s42003-019-0674-1)
Supplement: Supplementary file 2 — Description of Additional Supplementary Files [file 42003_2019_674_MOESM2_ESM.docx]

**Description of Additional Supplementary Files**

File Name: Supplementary Data 1

Description: The raw data for the generation of graphs in Figures 1-4 and Supplementary Figures 1-4.
