# Supplementary material for: Light-driven activation of mitochondrial proton-motive force improves motor behaviors in a Drosophila model of Parkinson’s disease
Source: Commun Biol. 2019 Nov 22;2:424. doi: 10.1038/s42003-019-0674-1 (PMC6874642; doi:10.1038/s42003-019-0674-1)
Supplement: Supplementary file 4 — Reporting Summary [file 42003_2019_674_MOESM4_ESM.pdf]

nature research

Corresponding author(s): Yuzuru Imai and Nobutaka Hattori

Last updated by author(s): Oct 20, 2019

Reporting Summary

Nature Research wishes to improve the reproducibility of the work that we publish. This form provides structure for consistency and transparency in reporting. For further information on Nature Research policies, see Authors & Referees and the Editorial Policy Checklist.

Statistics

For all statistical analyses, confirm that the following items are present in the figure legend, table legend, main text, or Methods section.

n/a	Confirmed	
☐	☒	The exact sample size (*n*) for each experimental group/condition, given as a discrete number and unit of measurement
☐	☒	A statement on whether measurements were taken from distinct samples or whether the same sample was measured repeatedly
☐	☒	The statistical test(s) used AND whether they are one- or two-sided<br>*Only common tests should be described solely by name; describe more complex techniques in the Methods section.*
☒	☐	A description of all covariates tested
☒	☐	A description of any assumptions or corrections, such as tests of normality and adjustment for multiple comparisons
☐	☒	A full description of the statistical parameters including central tendency (e.g. means) or other basic estimates (e.g. regression coefficient) AND variation (e.g. standard deviation) or associated estimates of uncertainty (e.g. confidence intervals)
☐	☒	For null hypothesis testing, the test statistic (e.g. *F*, *t*, *r*) with confidence intervals, effect sizes, degrees of freedom and *P* value noted<br>*Give P values as exact values whenever suitable.*
☒	☐	For Bayesian analysis, information on the choice of priors and Markov chain Monte Carlo settings
☒	☐	For hierarchical and complex designs, identification of the appropriate level for tests and full reporting of outcomes
☒	☐	Estimates of effect sizes (e.g. Cohen's *d*, Pearson's *r*), indicating how they were calculated

Our web collection on statistics for biologists contains articles on many of the points above.

Software and code

Policy information about availability of computer code

Data collection	All softwares used here have been listed in the Methods section.
Data analysis	All softwares we used in this study have been listed in the Methods section.

For manuscripts utilizing custom algorithms or software that are central to the research but not yet described in published literature, software must be made available to editors/reviewers. We strongly encourage code deposition in a community repository (e.g. GitHub). See the Nature Research guidelines for submitting code & software for further information.

Data

Policy information about availability of data

All manuscripts must include a data availability statement. This statement should provide the following information, where applicable:

- Accession codes, unique identifiers, or web links for publicly available datasets
- A list of figures that have associated raw data
- A description of any restrictions on data availability

The full data supporting this article are available from the corresponding author upon reasonable request. Source data are provided as a Source Data file.

Field-specific reporting

Please select the one below that is the best fit for your research. If you are not sure, read the appropriate sections before making your selection.

☒ Life sciences ☐ Behavioural & social sciences ☐ Ecological, evolutionary & environmental sciences

October 2018

Life sciences study design

All studies must disclose on these points even when the disclosure is negative.

Sample size	At least three independent experiments were performed for each data except for Fig S4c.
Data exclusions	We excluded data more than "mean ± standard division*2" in Fig. 1e, 3a and Supplementary Fig. 3c because live-imaging data are subjected to uncontrollable factors.
Replication	Western blot experiments were repeated at least three times.
Randomization	Randomization was performed.
Blinding	Abnormal mitochondria (Fig. 2h) and DA neurons (Fig. 4a,d) were counted and graded in a blinded manner by TI and HM. Blinding was not performed in other experiments.

Reporting for specific materials, systems and methods

We require information from authors about some types of materials, experimental systems and methods used in many studies. Here, indicate whether each material, system or method listed is relevant to your study. If you are not sure if a list item applies to your research, read the appropriate section before selecting a response.

Materials & experimental systems

n/a	Involved in the study	
☐	☒	Antibodies
☐	☒	Eukaryotic cell lines
☒	☐	Palaeontology
☐	☒	Animals and other organisms
☒	☐	Human research participants
☒	☐	Clinical data

Methods

n/a	Involved in the study	
☒	☐	ChIP-seq
☒	☐	Flow cytometry
☒	☐	MRI-based neuroimaging

Antibodies

Antibodies used	Anti-dCHCHD2 (1:1,000 dilution; in-house (Meng et al., 2017)), anti-Myc (1:1,000; Millipore, clone 4A6), anti-Tim23 (1:2,000; BD, clone 32/Tim23), anti-GAPDH (1:2,000; Bioss, clone 3E12), anti-actin (1:10,000; Millipore, clone C4), anti-NDUFS3 (1:2,000; Abcam, clone 17D95), anti-SDHA (1:1,000; GeneTex, GTX101689), anti-UQCRC1 (1:1,000; ThermoFisher Scientific, clone 11A51H12), anti-COX IV (1:1,000; Abcam, clone 20E8C12), anti-ATP5A (1:20,000; Abcam, clone 15H4C4), anti-4-HNE (1:500; JaICA, clone HNEJ-2), anti-alpha-synuclein (1:500; Abcam, clone MJFR1 and 1:500; BD Biosciences, clone 42/α-synuclein), anti-polyubiquitin (1:200; MBL, clone FK2), and anti-dTH (1:250; in-house (Yang et al., 2006))
Validation	Anti-dCHCHD2, anti-actin, anti-CoX IV and anti-4HNE were reported in Meng et al., 2017.<br>Anti-NDUFS3 and anti-ATP5A were used in Pimenta de Castro et al., 2012.<br>Anti-GAPDH were reported in Inoshita et al., 2017.<br>Anti-dTH were reported in Meng et al., 2017 and Yang et al 2006.<br>Anti-anti-polyubiquitin was used in Demontis and Perrimon 2010.<br>Anti-alpha-synuclein has been validated by many other studies.<br>Validations for anti-SDHA, anti-UQCRC1 and anti-Tim23 are based on the datasheets from the manufactures and their specific staining patterns in the literature.

Eukaryotic cell lines

Policy information about cell lines

Cell line source(s)	S2R+ cell line was obtained from the establisher Dr. N. Yanagawa at Kyoto University.
Authentication	S2R+ cell line was obtained from the establisher Dr. N. Yanagawa at Kyoto University.
Mycoplasma contamination	All the cells were tested and certified as mycoplasma-free.
Commonly misidentified lines (See ICLAC register)	None of the cell lines used is listed as commonly misidentified.

Animals and other organisms

Policy information about studies involving animals; ARRIVE guidelines recommended for reporting animal research

Laboratory animals	Drosophila melanogaster
Wild animals	The study did not involve wild animals.
Field-collected samples	The study did not involve samples collected from the field.
Ethics oversight	No ethical approval was not required for researches using insects.

Note that full information on the approval of the study protocol must also be provided in the manuscript.
